# Anion-Driven Circularly Polarized Luminescence Inversion of Unsymmetrical Europium(III) Complexes for Target Identifiable Sensing

**DOI:** 10.1021/acs.inorgchem.2c02202

**Published:** 2022-09-15

**Authors:** Yoshinori Okayasu, Kota Wakabayashi, Junpei Yuasa

**Affiliations:** Department of Applied Chemistry, Tokyo University of Science, 1-3 Kagurazaka, Shinjuku-ku, Tokyo 162-8601, Japan

## Abstract

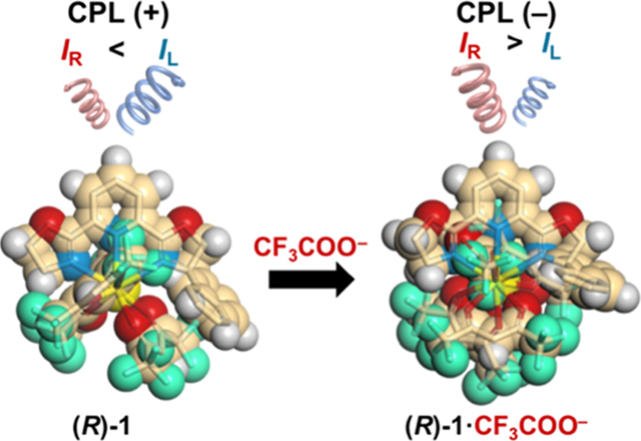

Anion-responsive sign inversion of circularly polarized luminescence (CPL) was successfully achieved by N_3_O_6_-type nona-coordinated europium(III) (Eu^3+^) complexes [(*R*)-**1** and (*S*)-**1**] composed of a less-hindered unsymmetrical N_3_-tridentate ligand (a chiral bis(oxazoline) ligand) and three O_2_-chelating (β-diketonate) ligands. Here, (*R*)-**1** exhibited a positive CPL signal (*I*_L_ – *I*_R_ > 0) at the ^5^D_0_ → ^7^F_1_ transition of Eu^3+^, which can be changed to a negative sign (i.e., *I*_L_ – *I*_R_ > 0 → *I*_L_ – *I*_R_ < 0) by the coordination of trifluoroacetic anions (CF_3_COO^–^) to the Eu^3+^ center. However, (*R*)-**1** preserved the original positive CPL signal (i.e., *I*_L_ – *I*_R_ > 0 → *I*_L_ – *I*_R_ > 0) in the presence of a wide range of competing anions (Cl^–^, Br^–^, I^–^, BF_4_^–^, ClO_4_^–^, ReO_4_^–^, PF_6_^–^, OTf^–^, and SbF_6_^–^). Thus, (*R*)-**1** acts as a smart target identifiable probe, where the CPL measurement (*I*_L_ – *I*_R_) can distinguish the signals from the competing anions (i.e., *I*_L_ – *I*_R_ < 0 vs *I*_L_ – *I*_R_ > 0) and eliminate the background emission (i.e., *I*_L_ – *I*_R_ = 0) from the background emitter (achiral luminescent compounds). The presented approach is also promising in terms of bio-inspired optical methodology because it enables nature’s developed chiral sensitivity to use circularly polarized light for object identification (i.e., *I*_L_ – *I*_R_ = 0 vs | *I*_L_ – *I*_R_ | > 0).

## Abstract 2

A N_3_O_6_-type nona-coordinated europium(III) (Eu^3+^) complex, (*R*)-**1** exhibited a positive CPL signal (*I*_L_ − *I*_R_ > 0) at the ^5^D_0_ → ^7^F_1_ transition of Eu^3+^, which can be changed to a negative sign (i.e., *I*_L_ − *I*_R_ > 0 → *I*_L_ − *I*_R_ < 0) by the coordination of trifluoroacetic anion (CF_3_COO^−^) to the Eu^3+^ center.

## Introduction

Molecular chirality is the origin of several chiroptical phenomena.^1^ Notably, circularly polarized luminescence (CPL) is a chiroptical phenomenon that is fascinating because of the potential usefulness of its chiroptical left- and right-handedness for information carriers,^2−15^ security,^16−18^ sensors,^19−33^ and displays.^34−37^ For developing these photo-technological applications using the chiroptical handedness of these molecules, the manipulation of the CPL sign by a simple input has become a focus of current research in photochemistry.^38,39^ Furthermore, the manipulation of the chiroptical materials exhibiting a large difference between left and right CPL intensity (*I*_L_ – *I*_R_) is of interest for potential applications to circularly polarized light for object identification (*vide infra*, *I*_L_ – *I*_R_ = 0 vs |*I*_L_ – *I*_R_| > 0).^40,41^ In this context, chiral europium (Eu^3+^) systems often exhibit a high level of luminescence dissymmetry (*g*_CPL_ = 2(*I*_L_ – *I*_R_)/(*I*_L_ + *I*_R_))^42−55^ because the ^5^D_0_ → ^7^F_1_ transition satisfies the magnetic-dipole selection rule, *J* = 0, ±1 (except 0 ↔ 0).^55,56^ Thus, the Eu^3+^-based chiroptical switch is a promising candidate for achieving CPL inversion with a high luminescence dissymmetry factor. However, Eu^3+^-based chiroptical switches are still extremely rare.^57−63^ Especially when one tries to use a weak guest anion as a chemical input, the relative inaccessibility of the Eu^3+^ metal center, which is blocked by several ligands, renders the chiroptical switch extraordinarily difficult to achieve.

**Scheme 1:**
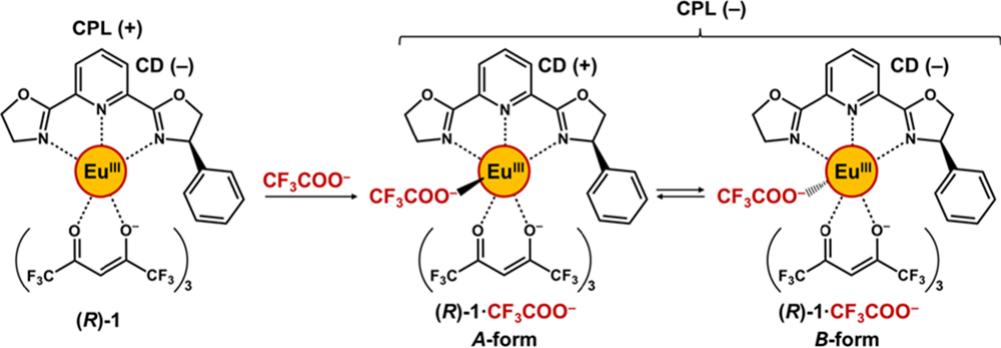
Scheme for CPL Sign Inversion of (*R*)-**1** upon Addition of CF_3_COO^–^ The CPL and CD signs correspond to those at the ^5^D_0_ → ^7^F_1_ transition of Eu^3+^ and the first Cotton band, respectively.

**Scheme 2:**
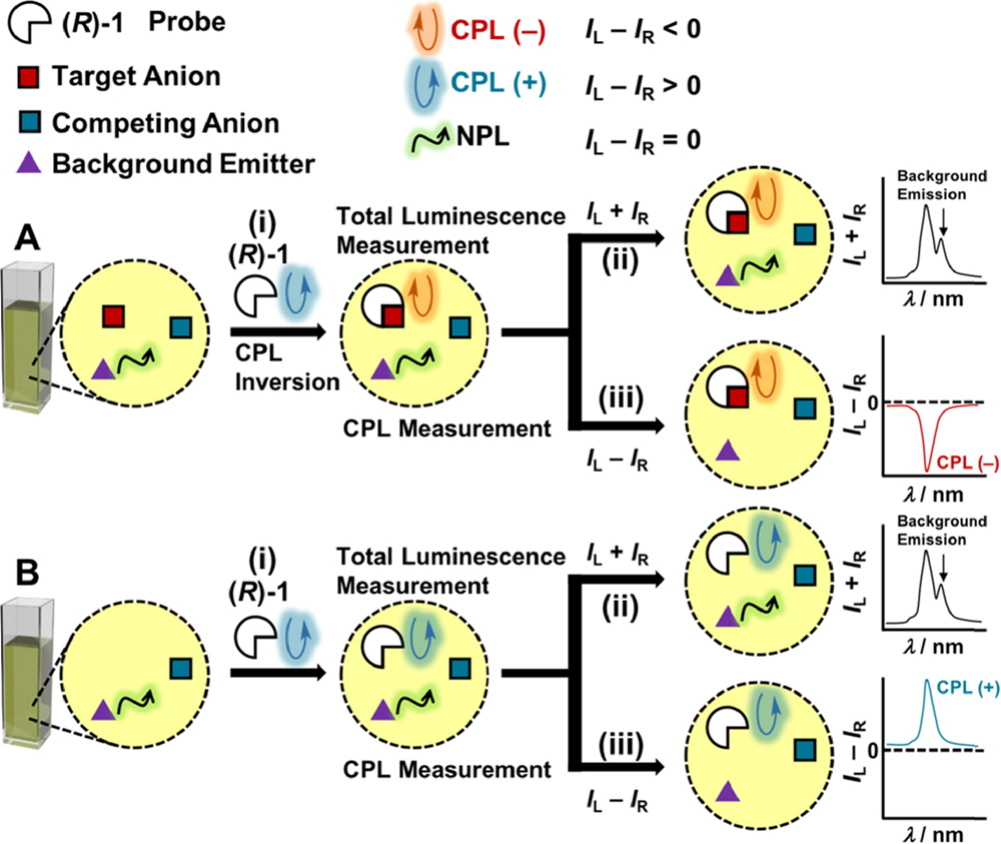
Schematic Representation of CPL Inversion-Used Anion Sensing for (A) Sample Containing Target Anion in Coexistence with the Competing Anion and a Background Emitter, (B) that in the Absence of Target Anion *I*_L_ and *I*_R_ denote the left and right CPL intensity, respectively.

Herein, for the first time, we report Eu^3+^ complexes [(*R*)-**1** and (*S*)-**1**] capable of switching the CPL sign in response to an anion guest [trifluoroacetic anion (CF_3_COO^–^)] and its use of target identifiable anion sensing. The present stimuli-responsive complexes have N_3_O_6_-type nona-coordinated structures^64−68^ composed of a chiral less-hindered unsymmetrical N_3_-tridentate ligand (L^*R*^ or L^*S*^)^69,70^ and three O_2_-chelating (β-diketonate) ligands. Coordination compounds with reduced symmetry, for which one can predict rationally the potential isomers from the combination of the unsymmetrical ligand and the metal ions, has garnered much interest among researchers in the field of coordination chemistry.^71−75^ In the present study, thanks to the sterically less-hindered position around the Eu^3+^ center, the three β-diketonate ligands are capable of rearrangement upon the binding of the anion to the Eu^3+^ center. The anion-binding event triggers sign inversion of the CPL of (*R*)-**1** at the ^5^D_0_ → ^7^F_1_ transition of Eu^3+^ (Scheme 1). Interestingly, the anion-responsive CPL inversion was observed only with CF_3_COO^–^, whereas (*R*)-**1** preserved the original positive CPL sign in the presence of a wide range of other competing anions (Cl^–^, Br^–^, I^–^, BF_4_^–^, ClO_4_^–^, ReO_4_^–^, PF_6_^–^, OTf^–^, and SbF_6_^–^). This response enables us to create a new CPL inversion-used target identifiable anion sensing probe illustrated by Scheme 2,^76−81^ which is inspired by naturally developed chiral sensitivity to use circularly polarized light for object identification (i.e., *I*_L_ – *I*_R_ = 0 vs |*I*_L_ – *I*_R_| > 0).^40,41^ When the CPL probe [(*R*)-**1**] is added to a solution containing both a target and its competing anions coexisting with a background emitter (Scheme 2A), the binding of (*R*)-**1** to the target anion causes the CPL sign inversion to give the negative CPL signal (i.e., *I*_L_ – *I*_R_ > 0 → *I*_L_ – *I*_R_ < 0). However, the original positive CPL signal of (*R*)-**1** remained unchanged in the absence of the target anion (i.e., *I*_L_ – *I*_R_ > 0 → *I*_L_ – *I*_R_ > 0, Scheme 2B). In such a case, the CPL analysis enables one to sense the target anion by using the sign of the CPL (i.e., *I*_L_ – *I*_R_ > 0 vs *I*_L_ – *I*_R_ < 0). Furthermore, the CPL measurement (*I*_L_ – *I*_R_) is also capable of eliminating the background emission from the background emitter (achiral luminescent compounds) that exhibits nonpolarized luminescence (NPL, *I*_L_ – *I*_R_ = 0) [Scheme 2iii]. Such CPL-driven object identification is never achieved by the total luminescence measurement (*I*_L_ + *I*_R_) normally used in fluorescence sensor systems [Scheme 2ii].^82−84^ In this context, we have recently reported CPL-driven cation (Zn^2+^) sensing by using pyrene-based fluorescence probes based on the cation-induced CPL turn-on mechanism (i.e., |*I*_L_ – *I*_R_| = 0 → |*I*_L_ – *I*_R_| > 0).^25−27^ Until the present, CPL-based anion sensing has been restricted mostly to chiral anion sensing, which can easily induce CPL signals on the probe emission (i.e., |*I*_L_ – *I*_R_| = 0 → |*I*_L_ – *I*_R_| > 0).^76,81,85,86^ To our knowledge, the present study is the first successful study demonstrating CPL-driven anion sensing based on the achiral anion-induced CPL sign inversion (i.e., *I*_L_ – *I*_R_ > 0 → *I*_L_ – *I*_R_ < 0). Thus, the beneficial effects of CPL on the present system will significantly expand the scope of the applications of photo-information technology as well as sensor technology.

## Results and Discussion

**Figure 1:**
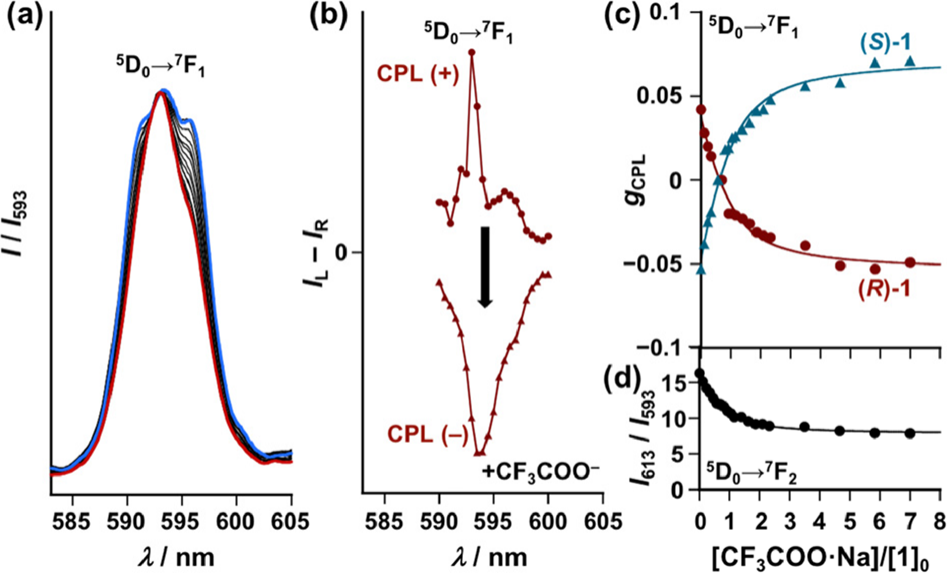
(a) Emission spectra (corresponding to ^5^D_0_ → ^7^F_1_) of (*R*)-**1** (1.0 × 10^–3^ M) in the presence of CF_3_COO·Na [0 (blue line)–7.0 × 10^–3^ M (red line)] in acetonitrile, where the emission intensity was normalized at λ_em_ = 593 nm. (b) CPL spectra (corresponding to ^5^D_0_ → ^7^F_1_) of (*R*)-**1** (1.0 × 10^–3^ M) in the absence (red circles) and presence (red triangles) of CF_3_COO·Na (7.0 × 10^–3^ M) in acetonitrile. Excitation wavelength: λ_ex_ = 305 nm. (c, d) Plots of (c) *g*_CPL_ at *λ* = 593 nm (corresponding to ^5^D_0_ → ^7^F_1_), (d) normalized emission intensity at λ = 613 nm (corresponding to ^5^D_0_ → ^7^F_2_) vs [CF_3_COO·Na]/[(*R*)- or (*S*)-**1**]_0_.

**Figure 2:**
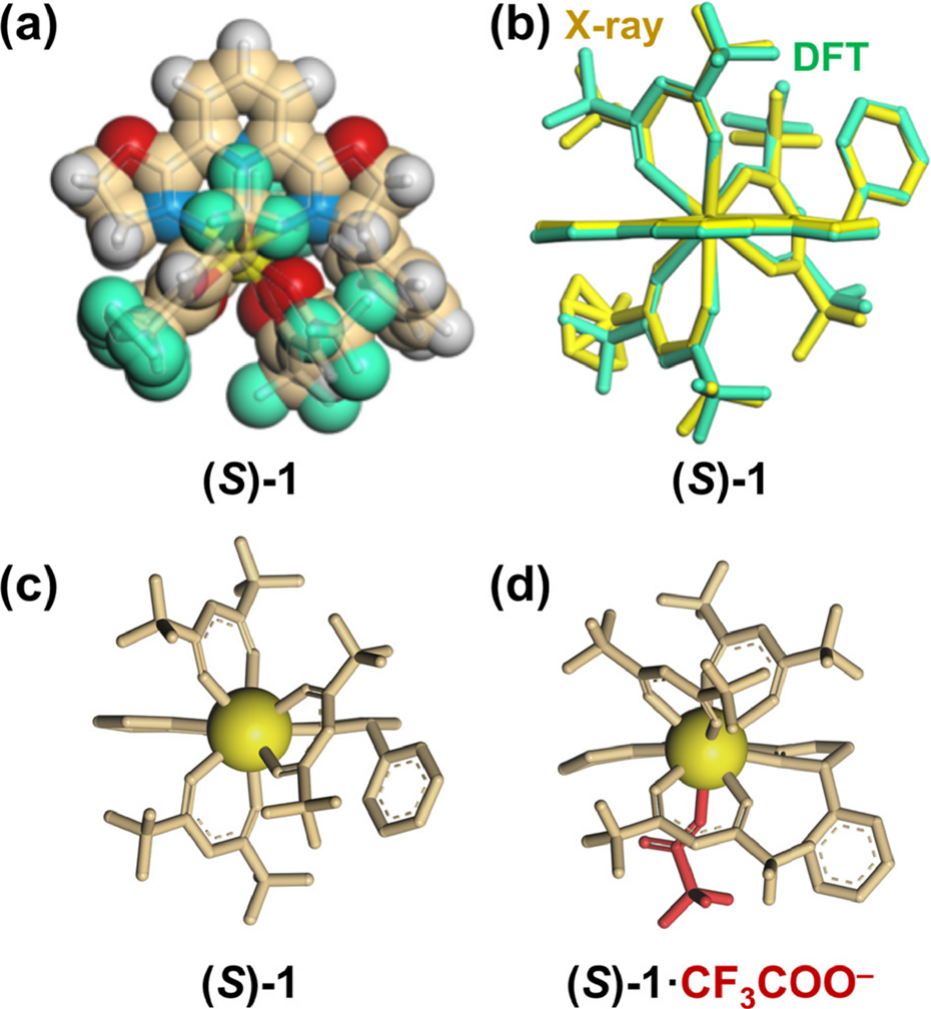
(a) X-ray crystal structure of (*S*)-**1** (CCDC 2097635). (b) Overlapping image of the X-ray crystal structure (yellow) of (*S*)-**1** and the optimized structure (green) [DFT/CAM-B3LYP/def2SVP (C H N O F)/def2TZVPP (La)] of (*S*)-**1**, (c, d) optimized structures [DFT/CAM-B3LYP/def2SVP (C H N O F)/def2TZVPP (La)] of (c) (*S*)-**1** and (d) (*S*)-**1**·CF_3_COO^–^ (*A*-form). For the DFT calculation, the Eu atoms were replaced by La atoms to reduce the calculation complexity. Hydrogen atoms are omitted for clarity (panel b–d).

The stimuli-responsive Eu^3+^ complexes [(*R*)-**1** and (*S*)-**1**] studied here were synthesized by reacting a tris-β-diketonate Eu^3+^ complex [Eu(HFA)_3_] with a less-hindered unsymmetrical bis(oxazoline) ligand (L^*R*^ or L^*S*^, respectively) in a 1:1 stoichiometry.^64−68^ The resulting complexes were characterized by X-ray structure analysis and electrospray ionization (ESI) mass spectrometry (*vide infra*; see details in the Supporting information). Figure 1a shows the CPL spectra of (*R*)-**1** at the ^5^D_0_ → ^7^F_1_ transition of Eu^3+^ before and after addition of the anion guest (CF_3_COO^–^) in acetonitrile. Before addition of the anion guest, (*R*)-**1** exhibited a positive CPL signal; however, (*R*)-**1** began to show a negative CPL signal after the addition of CF_3_COO·Na (Figure 1b, red circles to red triangles). Next, the *g*_CPL_ value at λ = 593 nm of (*R*)-**1** was plotted against the molar ratio [CF_3_COO·Na]/[(*R*)-**1**]_0_ (Figure 1c, red circles). Before the addition of the anion guest (i.e., [CF_3_COO·Na]/[(*R*)-**1**]_0_ = 0), (*R*)-**1** gave *g*_CPL_ = 0.042, which decreased with an increase in the molar ratio of the anion guest and reached a negative saturation value, *g*_CPL_ ∼ −0.05 (Figure 1c, red circles and Figure S1). Virtually, the mirror-image CPL inversion can be achieved with the enantiomer (*S*)-**1** (Figure 1c, blue triangles). A titration plot was also obtained from the emission intensity change at the ^5^D_0_ → ^7^F_2_ transition, which is sensitive to the coordination environment (Figures 1d and S1).^56,87^ With assuming a 1:1 association, the emission titration plot fits well the theoretical curve (Figure 1d), which allowed us to estimate a binding constant of (*R*)-**1** (and (*S*)-**1**) with CF_3_COO^–^ as (4.3 ± 0.6) × 10^3^ M^–1^. In the light of above results, we consider that coordination of the anion guest (CF_3_COO^–^) to the Eu^3+^ center of (*R*)-**1** (and (*S*)-**1**) is the trigger of the observed CPL sign inversion (Scheme 1). Conversely, coordination of the Na^+^ counter ion to the β-diketonate ligands might be an alternative mechanism for the CPL sign inversion. However, it should be noted that the Na^+^ cation with a noncoordinative counter anion (i.e., PF_6_·Na) had no effect on CPL or the emission spectrum of (*R*)-**1** (Figure S2). In contrast, the CF_3_COO^–^ anion with a different counter cation (i.e., CF_3_COO·NH_4_) was also capable of inducing the CPL sign inversion (Figure S3). Thus, the anion (CF_3_COO^–^) binding to the Eu^3+^ (Scheme 1) is the most probable mechanism of the present CPL sign inversion phenomena.^88^ Then, the possibility of the proposed anion-binding mechanism was considered based on the X-ray crystal structure of (*S*)-**1**. X-ray crystallography revealed that the less-hindered unsymmetrical N_3_-tridentate ligand (L^*S*^) creates a space for the three β-diketonate ligands to rearrange upon the anion-binding event (Figures 2a and S5). A tris-β-diketonate Eu^3+^ complex with a hindered symmetric N_3_-tridentate ligand having the two Ph side arms ((*R*)-**1′**)^64^ did not exhibit the CPL sign inversion even after addition of CF_3_COO^–^ (Figure S6), thus indicating the possible importance of the suggested space at the less-hindered position for the present CPL sign inversion mechanism. For further verification, the structure of (*S*)-**1** was optimized by density function theory (DFT)^89^ at CAM-B3LYP [def2SVP (C H N O F)/def2TZVPP (La)], which well reproduced the X-ray structure (Figure 2b). The observed good agreement (Figure 2b) suggests the validity of the DFT approach for the present system. Then, the 1:1 complex ((*S*)-**1**·CF_3_COO^–^) was also optimized by DFT to explore the coordination environment after the anion-binding event. The DFT-optimized structure of (*S*)-**1**·CF_3_COO^–^ suggests that the coordination of CF_3_COO^–^ causes huge impact on the positions of the three β-diketonate ligands (Figure 2c vs Figure 2d), which can affect the crystal field around the Eu^3+^ center. The CPL sign inversion (and emission spectral change) should be ascribed to such coordination rearrangement along with the anion-binding event (*vide infra*).

**Figure 3:**
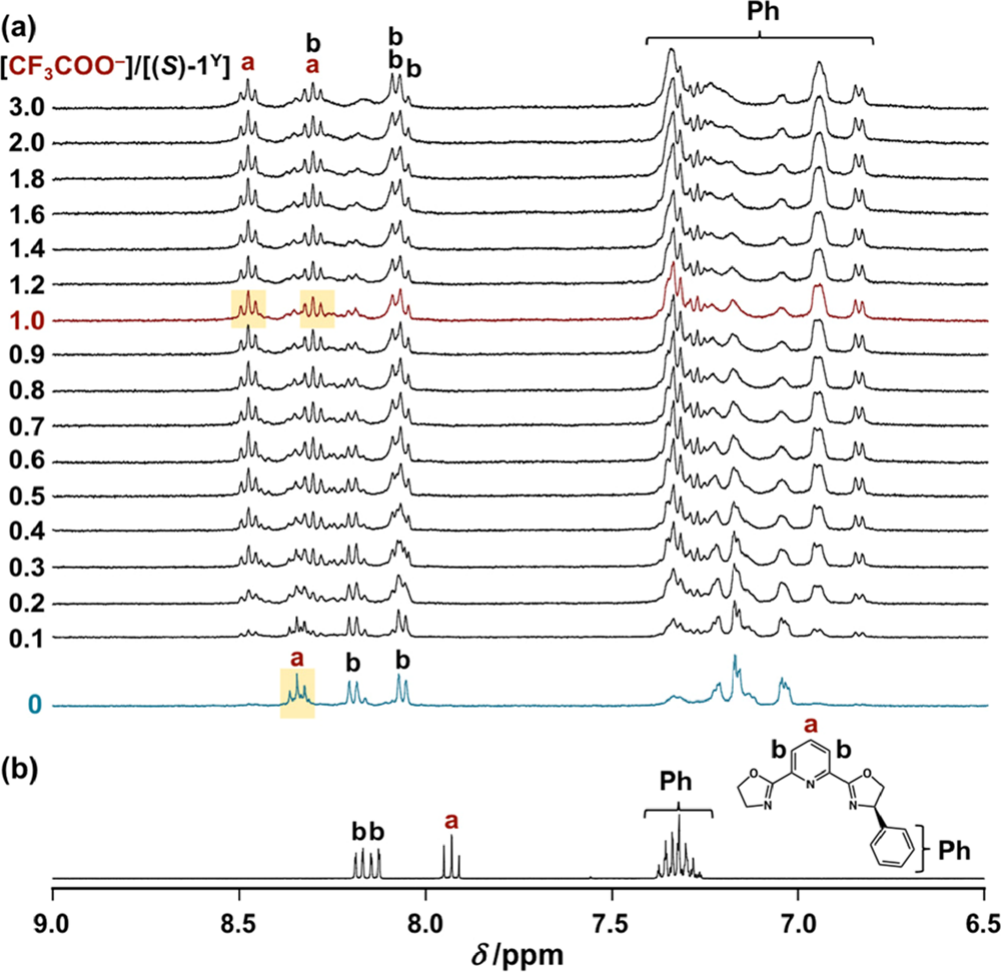
(a) Stacked ^1^H NMR spectra of (a) (*S*)-**1**^Y^ (2.0 × 10^–3^ M) in the presence of CF_3_COO·Na (0–6.0 × 10^–3^ M) in CD_3_CN at 298 K. (b) ^1^H NMR spectrum of L^*R*^ in CD_3_CN at 298 K. Signals corresponding to H^a^ are highlighted by yellow at [CF_3_COO^–^]/[(*S*)-**1**^Y^] = 0 and 1.0.

Next, the anion-binding event was investigated by ^1^H NMR titration, in which the paramagnetic Eu^3+^ ion was replaced by the diamagnetic yttrium ion (Y^3+^) to use (*S*)-**1**^Y^ as an isomorphous analog of (*S*)-**1**. Before the addition of CF_3_COO^–^, no signal splitting was observed in the pyridine proton at the 4-position (H^a^), suggesting that (*S*)-**1**^Y^ exists exclusively as a single diastereomer before the addition of CF_3_COO^–^ (Scheme 1). However, upon addition of 1.0 equiv of CF_3_COO^–^ (Figures 3a, red line and S7), the triplet signal (H^a^) splitted into two levels (as well as the other signals, some of which were overrated or broadening).^90^ No dissociated N_3_-tridentate chiral ligand was observed, even after the addition of CF_3_COO^–^ (Figure 3a vs Figure 3b). Thus, the observed signal splitting after the addition of CF_3_COO^–^ indicates the existence of two isomers (*A*- and *B*-forms) for (*S*)-**1**^Y^·CF_3_COO^–^ (*vide infra*, Scheme 1). The existence of two isomers was also indicated by emission lifetime data: in the presence of CF_3_COO^–^, (*R*)-**1** exhibited emission decay consisting of two components, τ = 1.50 ms (24%) and 0.89 ms (76%), while the emission of (*R*)-**1** had one component (τ = 0.99 ms) in the absence of CF_3_COO^–^ (Figure S9).^91^

**Figure 4:**
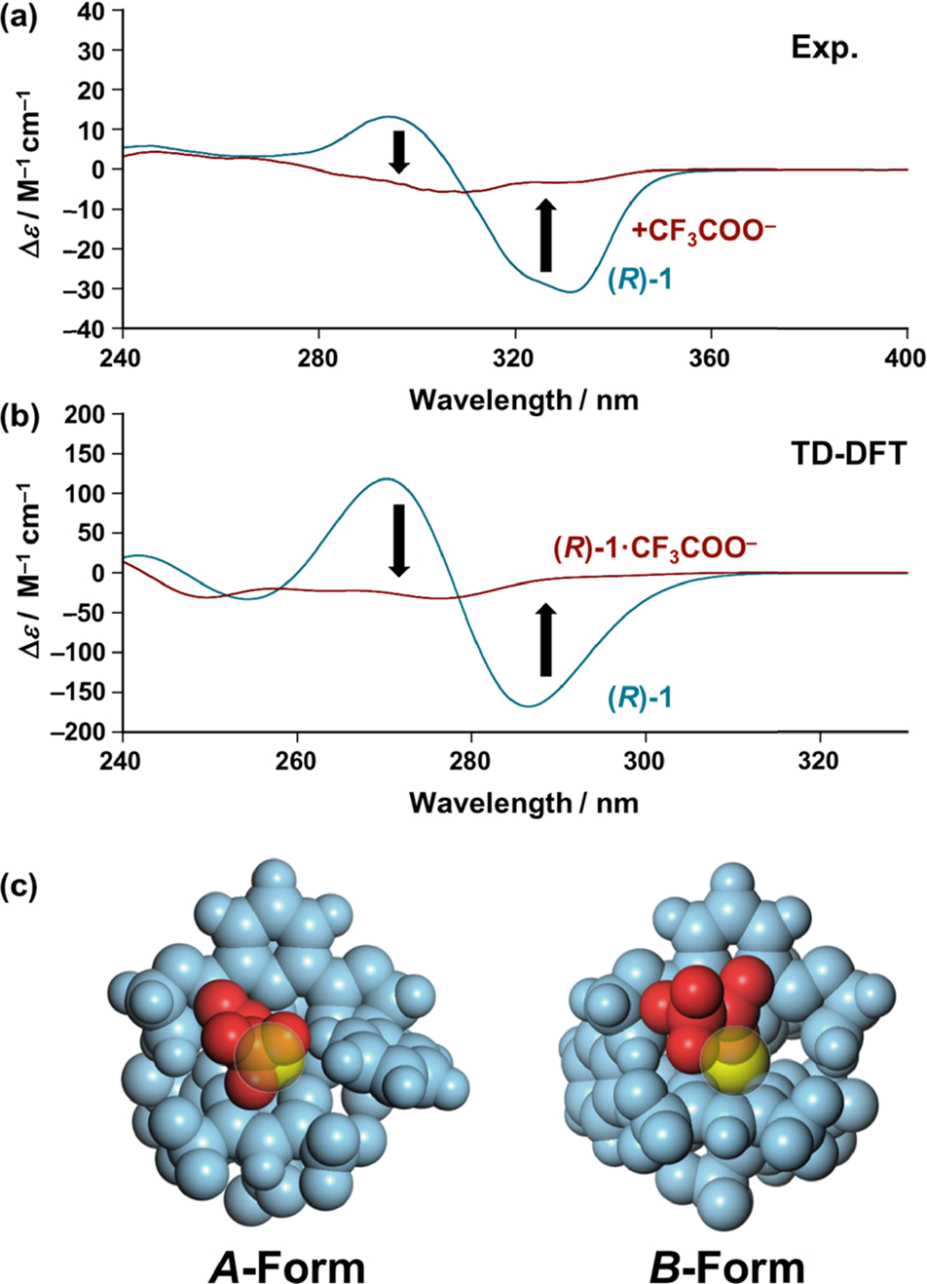
(a) CD spectra of (*R*)-**1** (1.0 × 10^–3^ M) in the absence (blue line) and presence (red line) of CF_3_COO·Na (7.0 × 10^–3^ M) in acetonitrile (1 mm path length quartz cell was used). (b) Theoretical CD spectrum [time dependent-DFT/CAM-B3LYP-6-31G(d) [C H N O F]/LANL2DZ (Sc)] of the optimized structure [DFT/CAM-B3LYP-6-31G(d) [C H N O F]/LANL2DZ (Sc)] of (*R*)-**1** (blue line) and (*R*)-**1**·CF_3_COO^–^ (red line) [*A*-form (35%), *B*-form (65%)], where Eu atoms are replaced by Sc atoms to reduce the calculation complexity (see details in Figure S10). (c) Optimized structures [DFT/CAM-B3LYP/def2SVP (C H N O F)/def2TZVPP (La)] of (*R*)-**1**·CF_3_COO^–^ (*A*-form and *B*-form), where the Eu atoms were replaced by La atoms to reduce the calculation complexity. Color code: CF_3_COO^–^ (red), La (yellow), ligands (blue).

In the light of these results, the rearrangement of the three β-diketonate ligands around the Eu^3+^ center upon the anion-binding event was monitored by using circular dichroism (CD) spectroscopy (Figure 4). Before the addition of CF_3_COO^–^, (*R*)-**1** exhibited a biphasic CD spectrum in the wavelength region of the β-diketonate ligand (Figure 4a, blue line), while the N_3_-tridentate chiral ligand (L^*R*^) had no appreciable absorption in this region. The characteristic biphasic CD bands arise from excitonic coupling between the β-diketonate ligands held in a chiral arrangement around the Eu^3+^ center, which can be well reproduced by time-dependent (TD) DFT (Figure 4b blue line; Figure S11).^75^ Conversely, upon addition of CF_3_COO^–^, both the negative and positive CD bands of (*R*)-**1** significantly decreased in intensity and essentially disappeared (Figure 4a, blue line to red line). The drastic change in the CD spectrum caused by CF_3_COO^–^ can be explained by considering the existence of the two competing isomers, as suggested by the above ^1^H NMR titration experiment (Figure 3, *vide supra*). Our DFT studies suggest that the two (diastereomer-like) isomers (*A*- and *B*-forms) of (*R*)-**1**·CF_3_COO^–^ have almost the same energy (Δ*E* = 0.06 kcal mol^–1^, Figure S12), whereas their theoretical CD spectra (TD-DFT) exhibit a quasi-mirror-image biphasic profile (Figure S13). Consequently, the resulting broad CD spectrum of (*R*)-**1** after the addition of CF_3_COO^–^ (Figure 4a, red line) was successfully reproduced by TD-DFT using the theoretical CD spectra of the *A*- and *B*-forms of (*R*)-**1**·CF_3_COO^–^ in a 35:65 ratio (Figure 4b, red line).^92^ These observations clarify that the anion-binding event induced the rearrangement of the three β-diketonate ligands around the Eu^3+^ center, which triggered the CPL sign inversion (Scheme 1).

**Figure 5:**
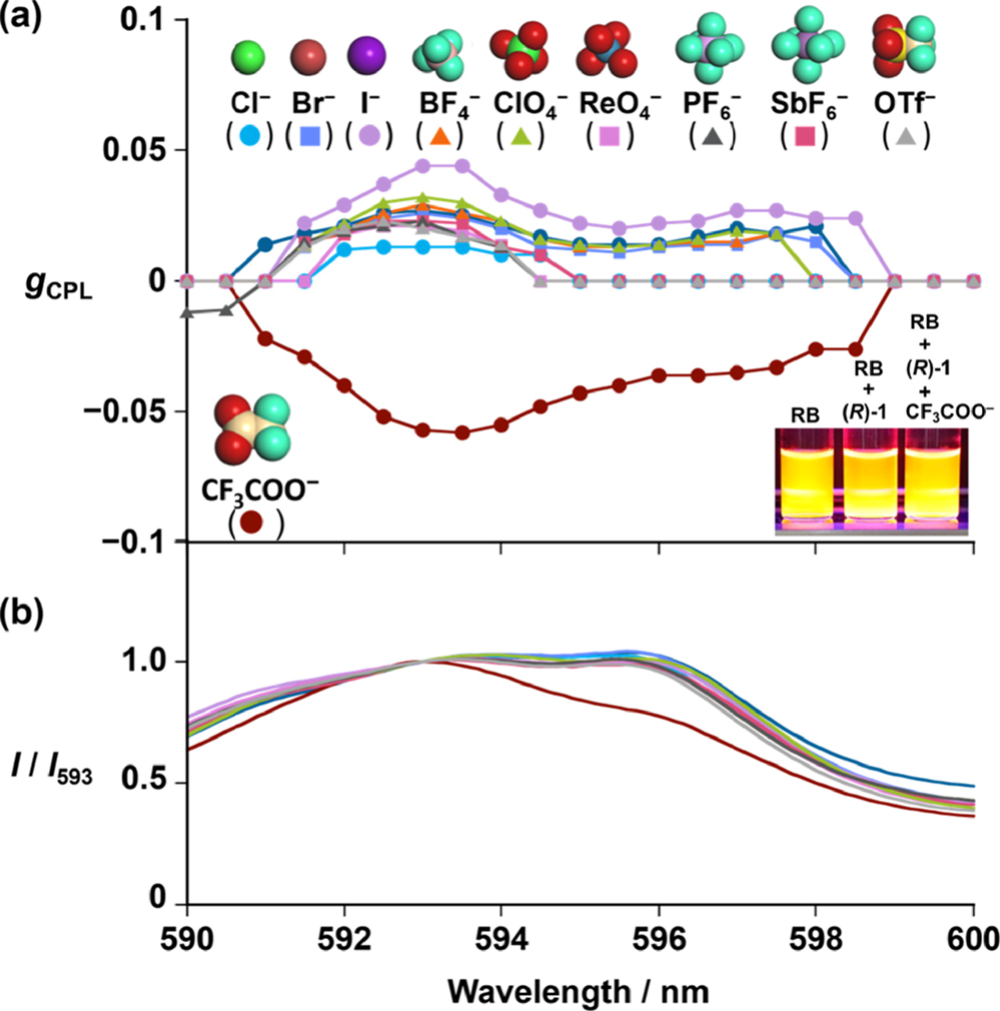
(a) CPL (*g*_CPL_) spectra of (*R*)-**1** in acetonitrile (1.0 × 10^–3^ M) containing rhodamine B (2.0 × 10^–4^ M) with the presence of the anion (concentration: 7.0 × 10^–3^ M) at 298 K, where *g*_CPL_ = 2(*I*_L_ – *I*_R_)/(*I*_L_ + *I*_R_). Tetrabutylammonium salts: Cl^–^, Br^–^, I^–^, BF_4_^–^, ClO_4_^–^, and ReO_4_^–^. Na^+^ salts: CF_3_COO^–^, PF_6_^–^, and OTf^–^. K^+^ salt: SbF_6_^–^. (b) Corresponding emission spectra. Excitation wavelength: λ_ex_ = 305 nm. Inset: (a) Visible emission photograph of acetonitrile solutions containing rhodamine B (RB) in the absence and presence of (*R*)-**1** and CF_3_COO^–^·Na^+^.

In light of the above results, we demonstrated the CPL inversion-used sensor performance of (*R*)-**1** for the target identifiable detection of the CF_3_COO^–^ anion across a wide range of competing anions (Cl^–^, Br^–^, I^–^, BF_4_^–^, ClO_4_^–^, ReO_4_^–^, PF_6_^–^, OTf^–^, and SbF_6_^–^)^93^ (Figure 5). Here, we used rhodamine B (RB) as a background emitter (achiral luminescent compounds) to test the object identification by CPL inversion-used anion sensing to eliminate the background luminescence (*vide supra*, Scheme 2). RB exhibits red emission similar to the Eu^3+^ emission; therefore, RB and Eu^3+^ emission (inset of Figure 5a) could not be distinguished visibly. In the same manner, we measured the emission and the CPL spectrum of (*R*)-**1** in the presence of various anions coexisting with RB (Figure 5a, b, respectively). The obtained emission spectrum of (*R*)-**1** with CF_3_COO^–^ (the target anion) and those with the competing anions suggested that the difference is relatively small and does not allow for clear differentiation of the anions (Figure 5b, red line for the target anion vs the other colored lines). Additionally, a broad emission band of RB overlapped with the Eu^3+^ emission band at the ^5^D_0_ → ^7^F_1_ transition in each case (Figure 5b). However, clear difference between CF_3_COO^–^ and the competing anions was obvious from CPL spectra: (*R*)-**1** preserved the original positive CPL sign (*I*_L_ – *I*_R_ > 0) toward the competing anions, whereas a negative CPL signal (*I*_L_ – *I*_R_ < 0) was successfully obtained only with CF_3_COO^–^ (Figure 5a). The lack of CPL sign inversion of (*R*)-**1** with the competing anions (Figure 5a) should be ascribed to weak or no interaction between the anions and (*R*)-**1** (Table S1 and Figure S14).^94,95^ Furthermore, the CPL measurement (Figure 5a) successfully eliminated the contribution of the background emitter (RB), which is CPL-silent (i.e., *I*_L_ – *I*_R_ = 0, Figure S15). The CPL-based CF_3_COO^–^ detection by (*R*)-**1** was successfully achieved even in coexistence with the competing anion OTf^–^ (Figure S16). Thus, (*R*)-**1** can detect the CF_3_COO^–^ anion identifiably by using the CPL signal as the detection output, eliminating the background emission (i.e., *I*_L_ – *I*_R_ = 0 vs *I*_L_ – *I*_R_ < 0) and distinguishing the target anion (CF_3_COO^–^) from the other competing anions (i.e., *I*_L_ – *I*_R_ < 0 vs *I*_L_ – *I*_R_ > 0), as illustrated by Scheme 2.

## Conclusions

In conclusion, we have successfully demonstrated that a less-hindered N_3_O_6_-type Eu^3+^ complex [(*R*)-**1**] is capable of switching the CPL handedness when triggered by binding of the trifluoroacetic anion (CF_3_COO^–^). The sign of the CPL signal of (*R*)-**1** remained unchanged in the presence of a wide range of competing anions. Such target anion-responsive CPL inversion of the signal of (*R*)-**1** can be successfully applied to CPL inversion-used anion sensing, in which the CPL measurement is capable of eliminating background emission and detecting CF_3_COO^–^. The presented approach is also interesting in terms of bio-inspired optical methodology because it enables nature’s developed chiral sensitivity to use circularly polarized light for object identification.^40,41^ In the future work, we will modify (such as solubility for various solvents) the Eu^3+^ complex to apply the present method for detection of a wide range of carboxylates (e.g., CH_3_COO^–^).

## Supporting Information Available

Experimental details; supporting titration experiments; suggested energy difference between the optimized structures; theoretical CD spectra; supporting emission spectra; emission lifetime data (PDF)
