# Supplementary material for: Anion-Driven Circularly Polarized Luminescence Inversion of Unsymmetrical Europium(III) Complexes for Target Identifiable Sensing
Source: Inorg Chem. 2022 Sep 15;61(38):15108–15. doi: 10.1021/acs.inorgchem.2c02202 (PMC9516667; doi:10.1021/acs.inorgchem.2c02202)
Supplement: Supplementary file 1 — ic2c02202_si_001.pdf [file ic2c02202_si_001.pdf]

# Supporting Information

## Anion-Driven Circularly Polarized Luminescence Inversion of Unsymmetrical Europium(III) Complexes for Target Identifiable Sensing

Yoshinori Okayasu, Kota Wakabayashi, and Junpei Yuasa*

*Department of Applied Chemistry, Tokyo University of Science, 1-3 Kagurazaka, Shinjuku, Tokyo 162-8601, Japan.; E-mail: yuasaj@rs.tus.ac.jp*

**Experimental Section**

**Chemicals and Reagents.** Chemicals were purchased from Wako Pure Chemical Industries Ltd. and used as received without further purification. 1,1,1,5,5,5-hexafluoroacetylacetone was purchased by TCI. (*R*)-2-(6-(4,5-dihydrooxazol-2-yl)pyridin-2-yl)-4-phenyl-4,5-dihydrooxazole was prepared according to a procedure described previously.[1]

**Materials and Methods.** JEOL ECZ400S (400 MHz) was used for $^{1}$H NMR studies. The Positive ESI mass spectra of the Eu(III) complexes were recorded at JEOL AccuTOF CS JMS-T100CS. The UV-vis absorption and emission spectra were measured by using JASCO V-660 and FP-6500, respectively. We measured CPL spectra of (*S*)-**1** and (*R*)-**1** by using a homemade CPL spectroscopy system.[2]

**Crystallography.** Suitable crystal of (*S*)-**1** (CCDC 2097635) was grown by slow evaporation of the methanol solution containing (*S*)-**1**. X-ray diffraction intensity was collected with a Bruker AXS・SMART APEX CCD detector with graphite monochromated Mo K$\alpha$ radiation at 173 K. APEX3 software was used for all calculations.

**Titration Experiments.** Titration experiments were conducted by successive addition of the stock solution containing the high concentrations of the guest anions to the solutions of (*S*)-**1** and (*R*)-**1**, where the concentrations of kept (*S*)-**1** and (*R*)-**1** constant. All experiments were performed at room temperature.

**Synthesis.** The precursor complexes (tris-$\beta$-diketonate $Eu^{3+}$ complexes) were synthesized according to the literature.[3] We synthesized (*S*)-**1** and (*R*)-**1** as follows. Typically, (*R*)-2-(6-(4,5-dihydrooxazol-2-yl)pyridin-2-yl)-4-phenyl-4,5-dihydrooxazole (0.17 mmol) and tris-$\beta$-diketonate $Eu^{3+}$ complex (0.17 mmol) were dissolved in 30 mL of methanol. Then, the reaction solution was stirred for overnight at room temperature. The white powder obtained by the solvent evaporation was dried under vacuum (yield: 85%). (*R*)-**1**: HRMS (ESI+) *m*/*z* calculated for $C_{32}H_{18}EuF_{18}N_3O_8Na^+$ $[M+Na]^+$ *m/z* = 1089.99164, found 1089.99466. (*S*)-**1**: HRMS [ESI-MS (positive)]: *m*/*z* calcd. for $C_{32}H_{18}EuF_{18}N_3O_8Na^+$ $[M+Na]^+$ *m/z* = 1089.99164, found 1089.98889. (*S*)-$\mathbf{1}^{\mathbf{Y}}$ was prepared by the same procedure as used for (*S*)-**1**: HRMS [ESI-MS (positive)]: *m*/*z* calcd. for $C_{32}H_{18}F_{18}N_3NaO_8Y^+$ $[M+Na]^+$ *m/z* = 1025.9757, found 1025.9758.

**Structure Modeling.** The optimized structures of metal complexes were calculated with GAUSSIAN 09.[4] For the optimized structure of (*S*)-**1**·$CF_3COO^-$, the initial input structure was modeled by coordination of $CF_3COO^-$ to the central La atom from the less hindered position.

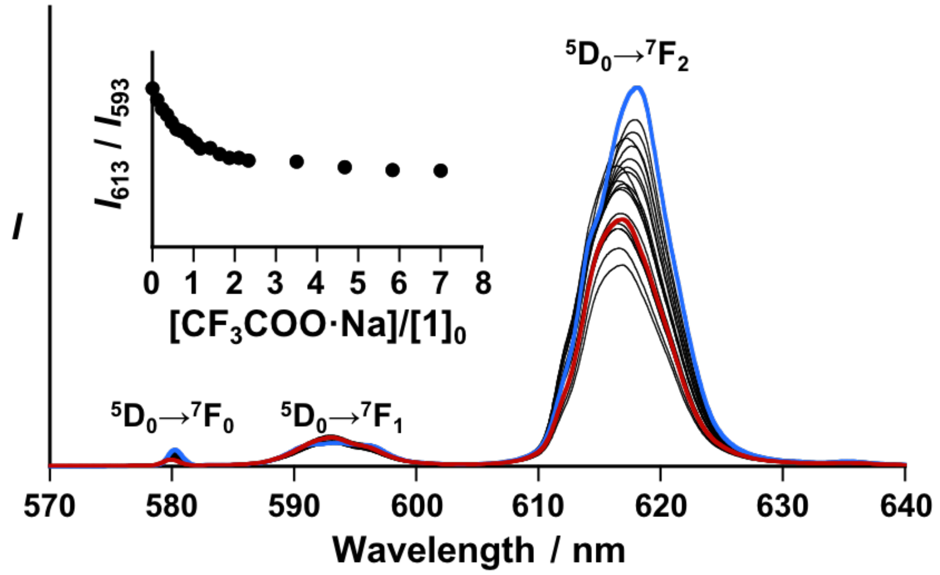


***Figure S1*****.** Emission spectra of (*R*)-**1** ($1.0 \times 10^{-3}$ M) in the presence of $CF_3COO$·Na [0 (blue line) – $7.0 \times 10^{-3}$ M (red line)] in acetonitrile. Excitation wavelength: $\lambda_{ex}$ = 305 nm.

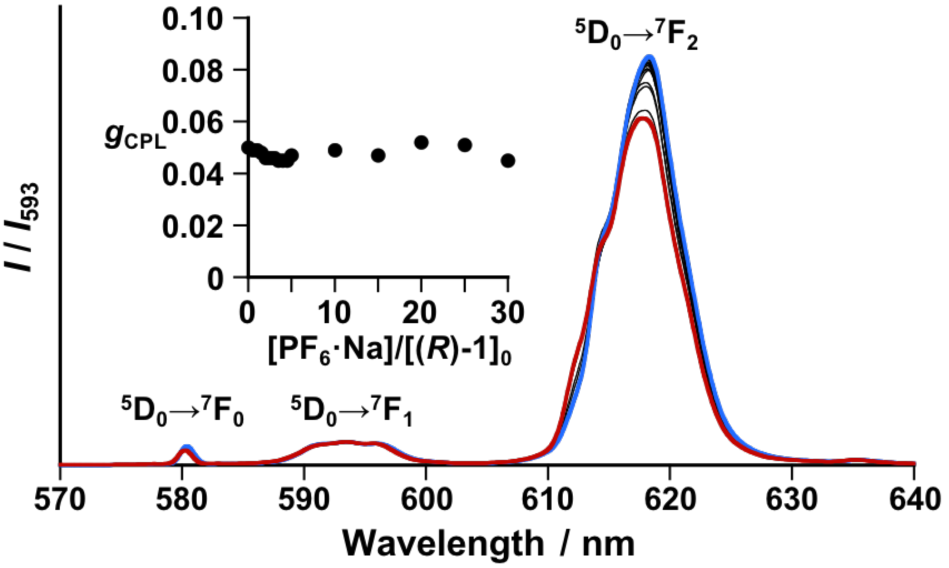


***Figure S2*****.** Emission spectra of (*R*)-**1** ($1.0 \times 10^{-3}$ M) in the presence of $PF_6$·Na [0 (blue line) – $3.0 \times 10^{-2}$ M (red line)] in acetonitrile, where emission intensity was normalized at $\lambda_{em}$ = 593 nm. Inset shows plot of $g_{CPL}$ at $\lambda$ = 593 nm versus [$PF_6$·Na]/[(*R*)-**1**]$_0$. Excitation wavelength: $\lambda_{ex}$ = 305 nm.

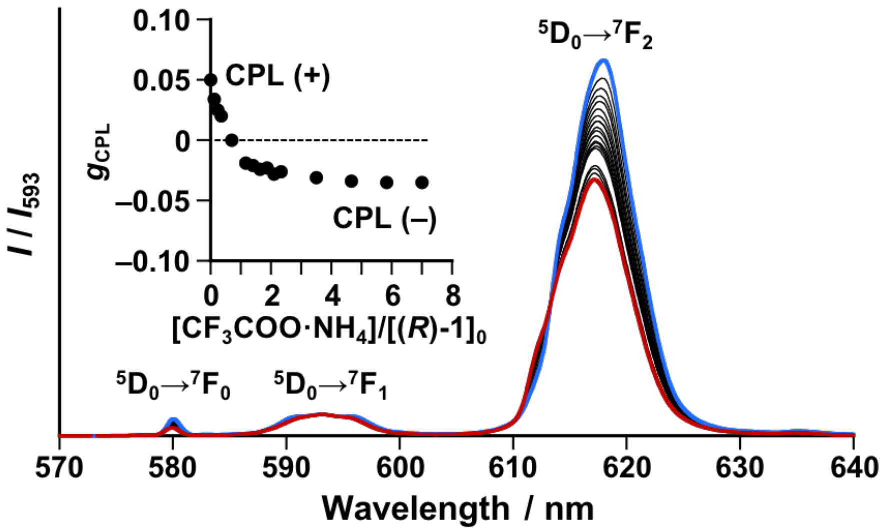


***Figure S3.*** Emission spectra of (*R*)-**1** ($1.0 \times 10^{-3}$ M) in the presence of $CF_3COO·NH_4$ [0 (blue line) – $7.0 \times 10^{-3}$ M (red line)] in acetonitrile, where emission intensity was normalized at $\lambda_{em}$ = 593 nm. Inset shows plot of $g_{CPL}$ at $\lambda$ = 593 nm versus [$CF_3COO·NH_4$]/[(*R*)-**1**]$_0$. Excitation wavelength: $\lambda_{ex}$ = 305 nm.

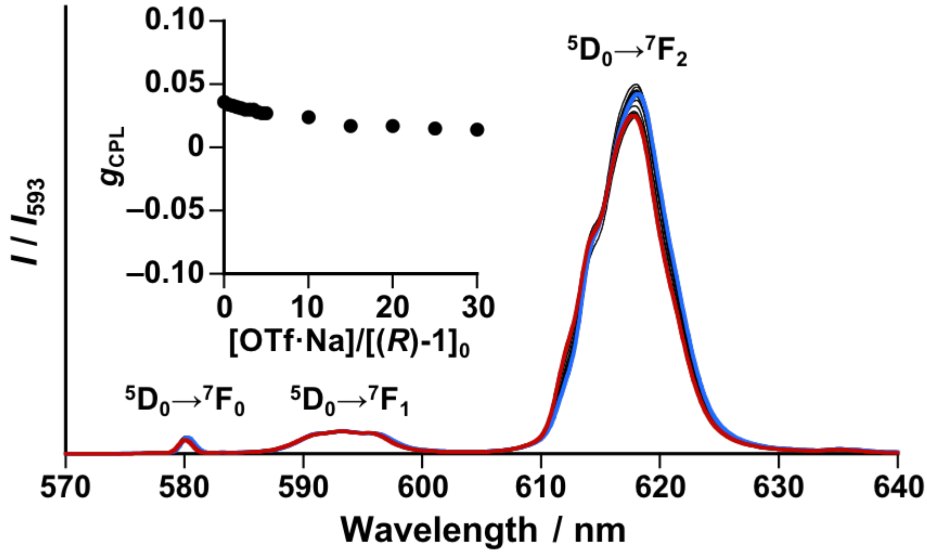


***Figure S4.*** Emission spectra of (*R*)-**1** ($1.0 \times 10^{-3}$ M) in the presence of OTf·Na [0 (blue line) – $3.0 \times 10^{-2}$ M (red line)] in acetonitrile, where emission intensity was normalized at $\lambda_{em}$ = 593 nm. Inset shows plot of $g_{CPL}$ at $\lambda$ = 593 nm versus [OTf·Na]/[(*R*)-**1**]$_0$. Excitation wavelength: $\lambda_{ex}$ = 305 nm.

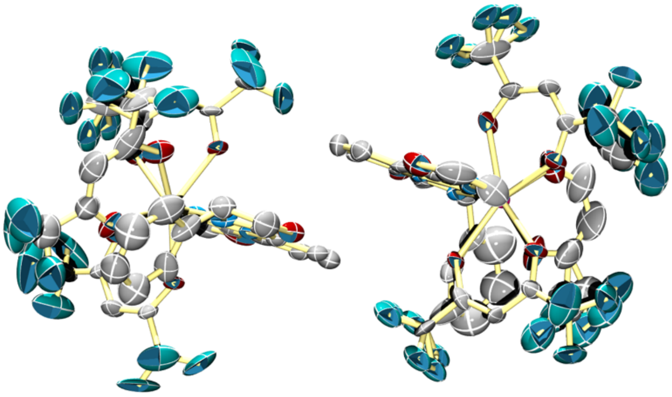

***Figure S5***. ORTEP view (50% probability) of (*S*)-**1**. Hydrogen atoms are omitted for clarity.

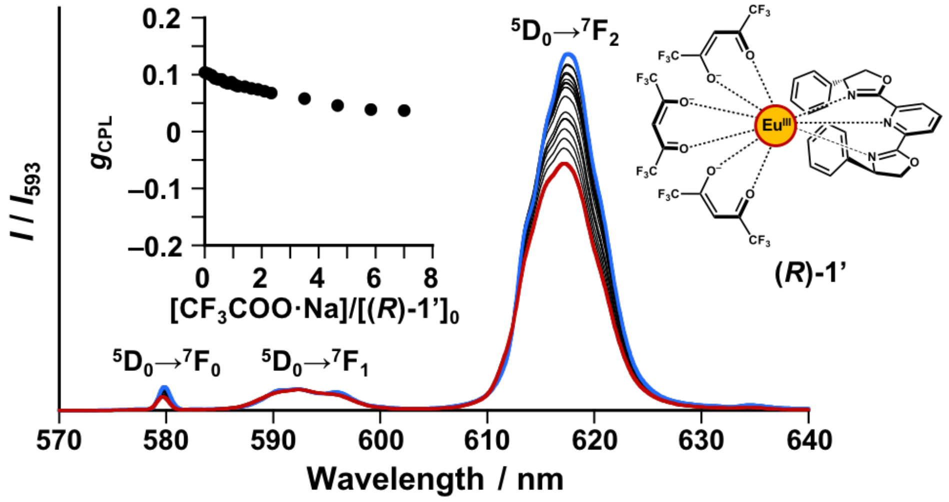


***Figure S6***. Emission spectra of (*R*)-**1'** ($1.0 \times 10^{-3}$ M) in the presence of $CF_3COO{\cdot}Na$ [0 (blue line) – $7.0 \times 10^{-3}$ M (red line)] in acetonitrile, where emission intensity was normalized at $\lambda_{em}$ = 593 nm. Inset shows plot of $g_{CPL}$ at $\lambda$ = 593 nm versus $[CF_3COO{\cdot}Na]/[(R)\text{-}\mathbf{1'}]_0$. Excitation wavelength: $\lambda_{ex}$ = 320 nm.

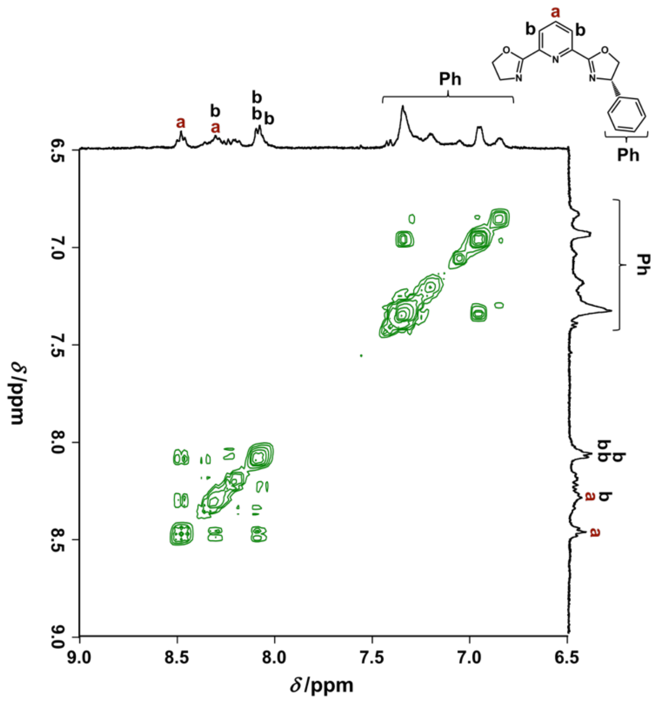


***Figure S7.*** $^1H,^1H$ COSY NMR spectrum of (*S*)-**1**$^{Y}$ ($2.0 \times 10^{-3}$ M) in the presence of $CF_3COO{\cdot}Na$ ($4.0 \times 10^{-3}$ M) in $CD_3CN$.

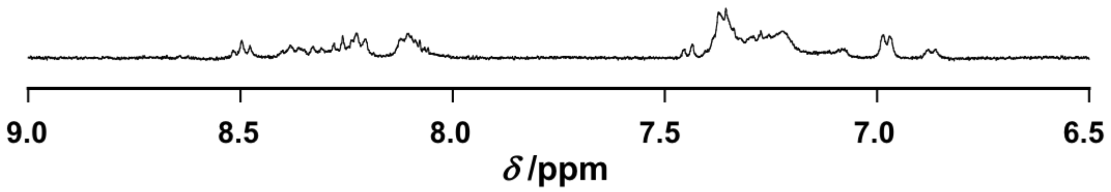


***Figure S8.*** $^1H$ NMR spectrum of (*S*)-**1**$^{Y}$ ($2.0 \times 10^{-3}$ M) in the presence of $CF_3COO{\cdot}Na$ ($2.0 \times 10^{-3}$ M) in $CD_3CN$ at 323 K.

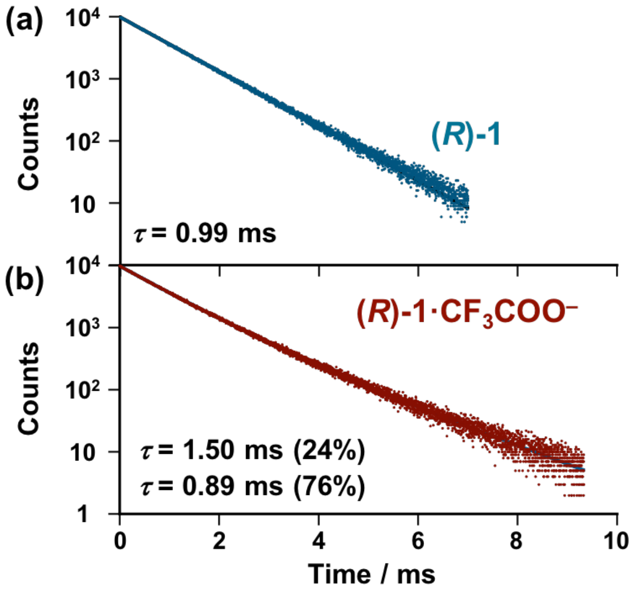


***Figure S9.*** Emission decay curves of (*R*)-**1** ($1.0 \times 10^{-3}$ M) (a) in the absence and (b) presence of $CF_3COO{\cdot}Na$ ($1.0 \times 10^{-3}$ M) in acetonitrile. Excitation wavelength: $\lambda_{ex}$ = 371 nm.

**With regard to the TD DFT calculations (S10, S11):** The DFT optimized structure of (*S*)-**1** well reproduced the X-ray crystal structure of (*S*)-**1**, where the Eu atoms were replaced by La atoms Conversely, the obtained DFT optimized structures could not be suitable for the subsequent time-dependent (TD) DFT calculations, where an error with regard to frozen core potential of La atoms was occurred. Consequently, we calculated alternative optimized structures by replacing the Eu atoms with Sc atoms [DFT/CAM-B3LYP-6-31G(d) [C H N O F]/LANL2DZ (Sc)]. This modification has no significant impact on the positions of the three $\beta$-diketonate ligands around the $Eu^{3+}$ center (Figure S10), while the two $L^S$ ligands underwent slight position changes. The position changes of $L^S$ have no significant impact on the theoretical CD spectra, since $L^S$ has no appreciable absorption band in the spectral range of the experimentally observed CD spectrum. Then, good reproducibility was successfully achieved between the experimental and theoretical CD spectra (Fig. 4). The observed biphasic CD profile is mainly attributed to the excitonic coupling between the $\beta$-diketonate ligands held in chiral arrangement around the $Eu^{3+}$ center (Figure S11), while electronic transition of the $N_3$-tridentate chiral ligand has no significant impact on the observed wavelength region.

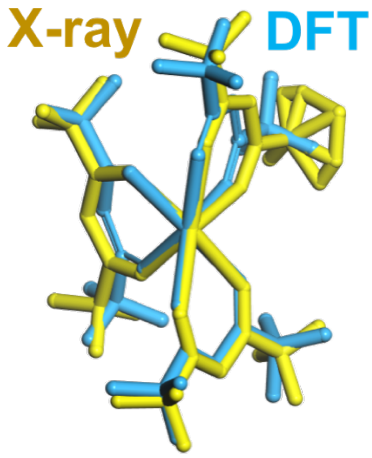


***Figure S10.*** Coordination arrangement of the three $\beta$-diketonate ligands found in X-ray crystal structure (yellow) and the optimized structure (blue) [DFT/CAM-B3LYP-6-31G(d) [C H N O F]/LANL2DZ (Sc)] of (*S*)-**1**, where Eu atoms are replaced by Sc atoms to reduce the calculation complexity. Hydrogen atoms are omitted for clarity.

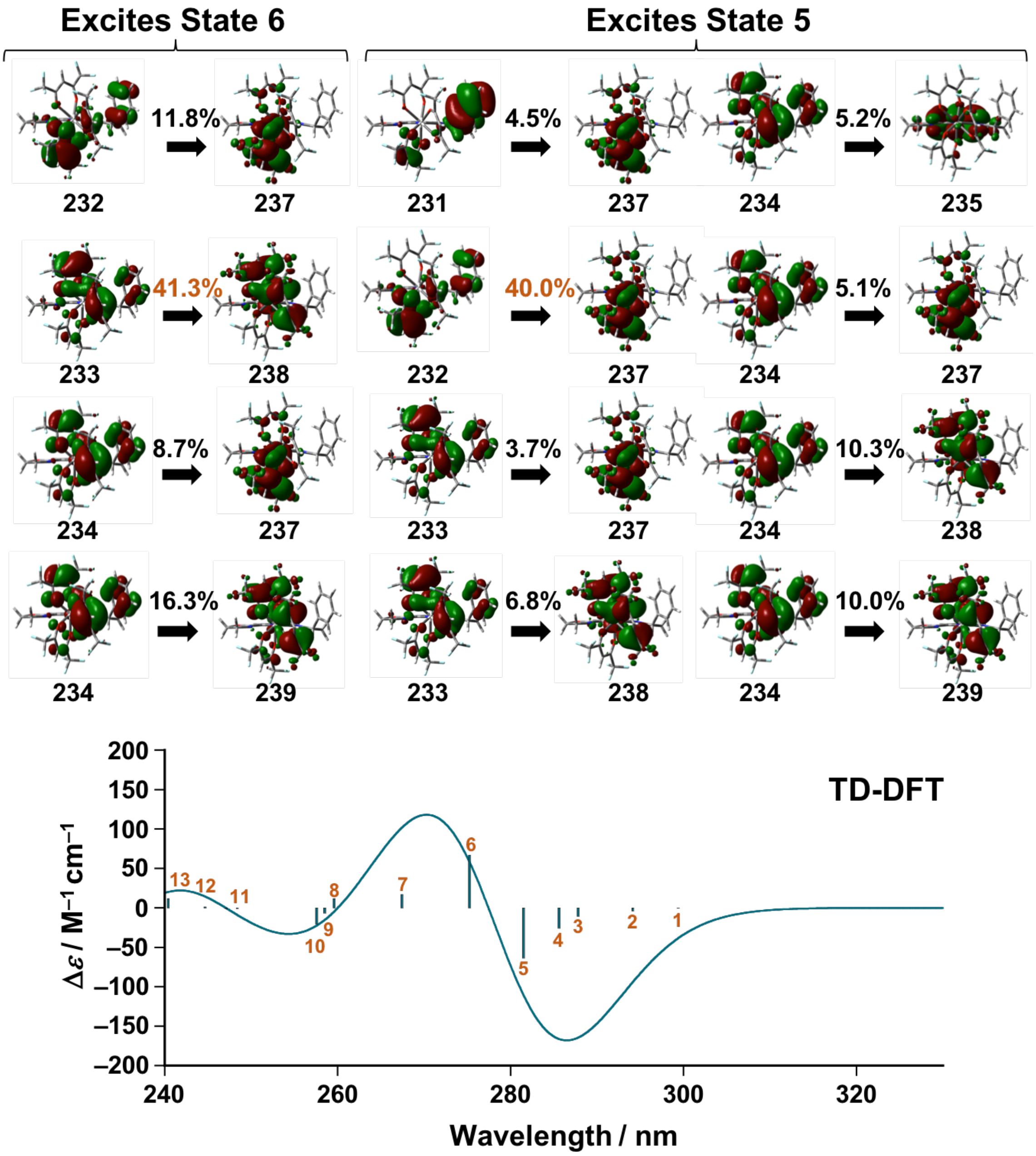


***Figure S11.*** Theoretical CD spectrum [time dependent-DFT/CAM-B3LYP-6-31G(d) [C H N O F]/LANL2DZ (Sc)] of the optimized structure [DFT/CAM-B3LYP-6-31G(d) [C H N O F]/LANL2DZ (Sc)] of (*R*)-**1** (blue line), where Eu atoms are replaced by Sc atoms to reduce the calculation complexity. Summary of excited state 5 and 6.

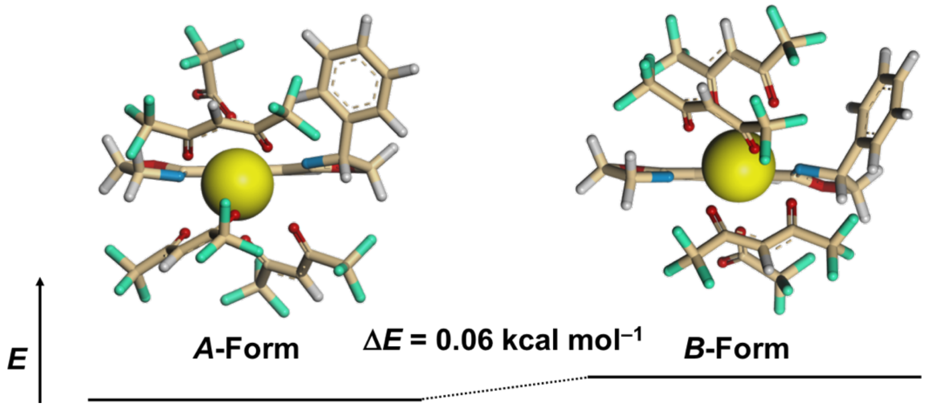


***Figure S12.*** Energy difference between the optimized structures [DFT/CAM-B3LYP/def2SVP (C H N O F)/def2TZVPP (La) in IEFPCM: acetonitrile] of (a) (*R*)-**1**·$CF_3COO^-$ (*A*-form) and (b) (*R*)-**1**·$CF_3COO^-$ (*B*-form), where the Eu atoms were replaced by La atoms to reduce the calculation complexity.

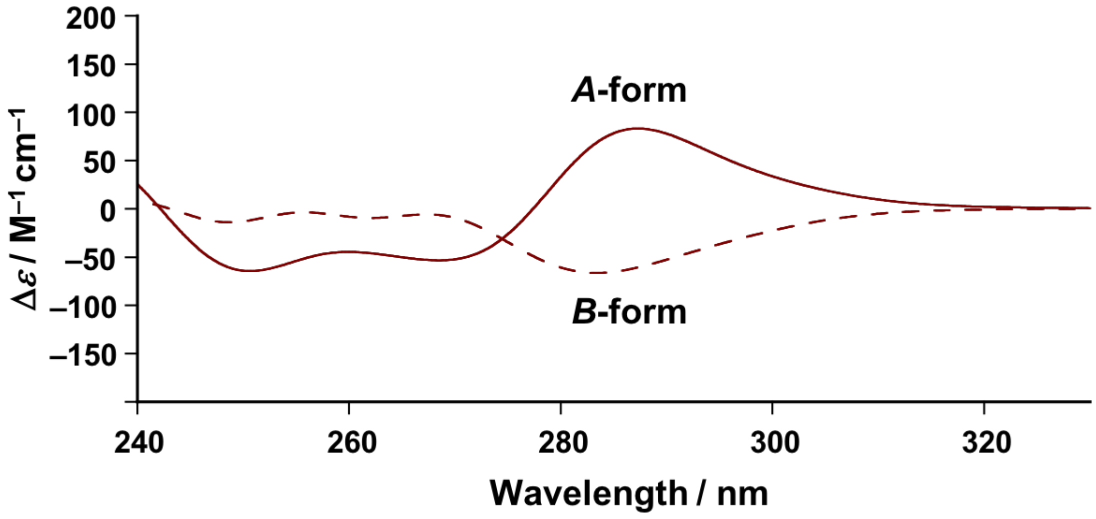


***Figure S13.*** Theoretical CD spectrum [time dependent-DFT/CAM-B3LYP-6-31G(d) [C H N O F]/LANL2DZ (Sc)] of the optimized structure [DFT/CAM-B3LYP-6-31G(d) [C H N O F]/LANL2DZ (Sc)] of (*R*)-**1**·$CF_3COO^-$ (*A*-form) [solid line] and (*R*)-**1**·$CF_3COO^-$ (*B*-form) [dashed line], where Eu atoms are replaced by Sc atoms to reduce the calculation complexity.

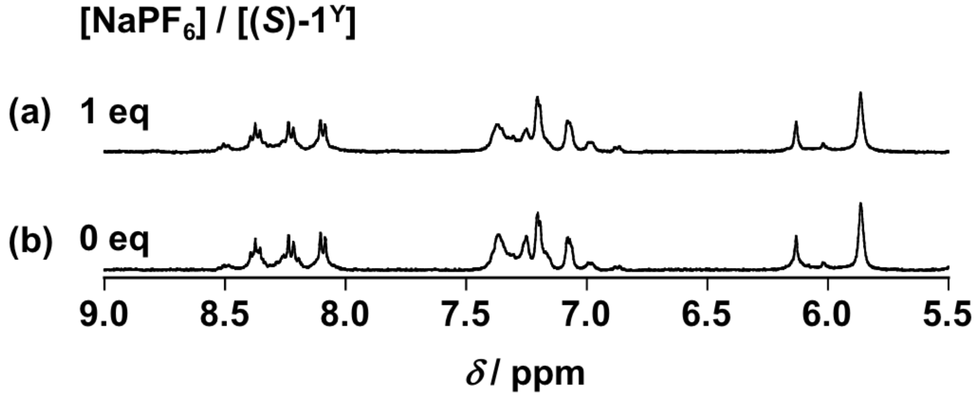


***Figure S14.*** $^1$H NMR spectra of (*S*)-**1**$^Y$ ($2.0 \times 10^{-3}$ M) (b) in the absence and (a) in the presence of $PF_6{\cdot}Na$ ($2.0 \times 10^{-3}$ M) in $CD_3CN$.

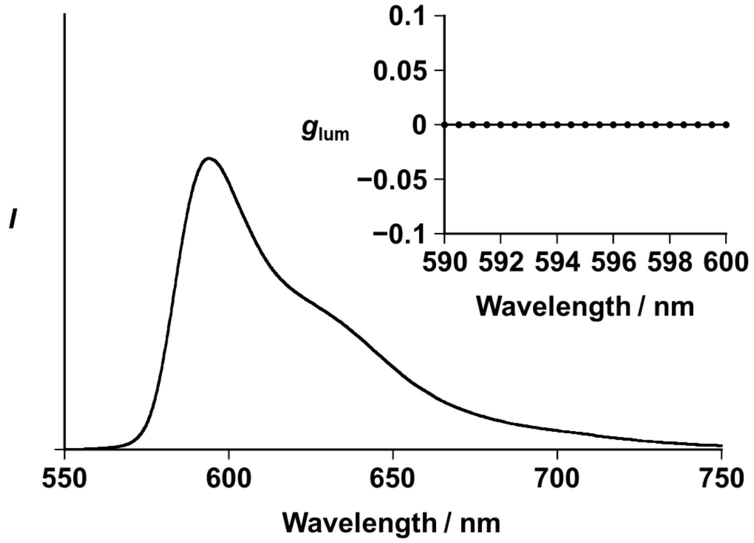


***Figure S15.*** Emission spectrum of rhodamine B in acetonitrile ($2.0 \times 10^{-4}$ M) at 298 K. Inset: Corresponding CPL ($g_{lum}$) spectrum. Excitation wavelength: $\lambda_{ex}$ = 305 nm.

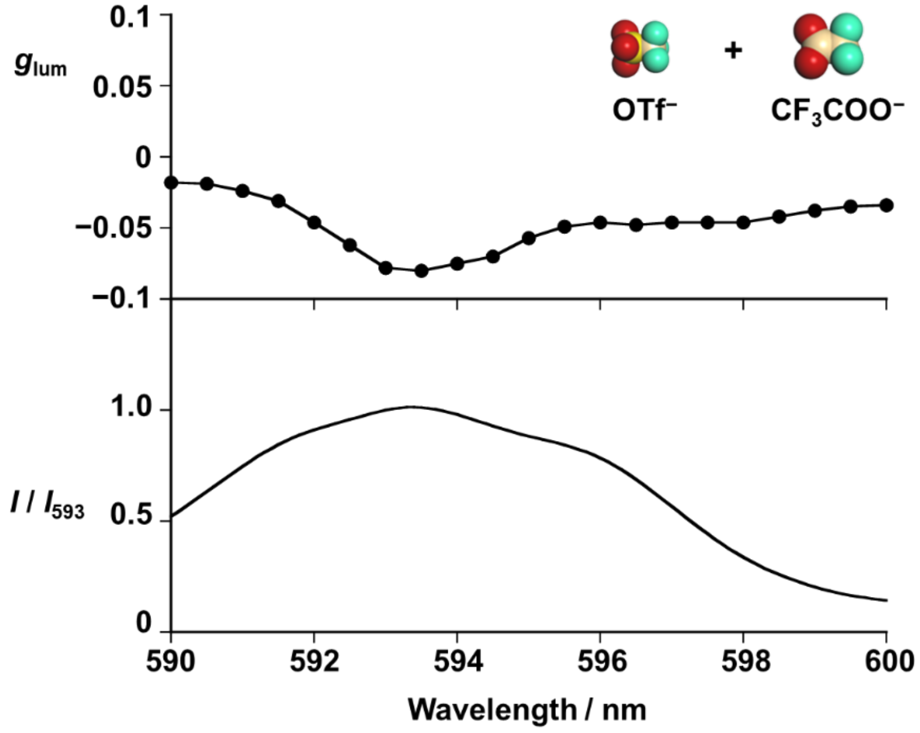


***Figure S16.*** (Top) CPL and (bottom) emission spectra of (*R*)-**1** in acetonitrile ($1.0 \times 10^{-3}$ M) with the presence of OTf·Na ($7.0 \times 10^{-3}$ M) and $CF_3COO$·Na ($7.0 \times 10^{-3}$ M) at 298 K. Excitation wavelength: $\lambda_{ex}$ = 305 nm.

**Table S1.** Summary of anion binding energy of (*R*)-**1** obtained with DFT

| 1:1 Complex | Optimized Structure DFT/CAM-B3LYP/def2SVP (C H N B O F Cl S P)/def2TZVPP (La Re Sb) | | Anion Binding Energy [a] (kcal mol$^{-1}$) | |
|---|---|---|---|---|
| | *A*-Form | *B*-Form | *A*-Form | *B*-Form |
| (*R*)-**1**·$BF_4^-$ | 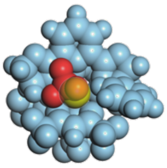 | 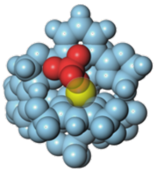 | 38.0 | 37.7 |
| (*R*)-**1**·$ClO_4^-$ | 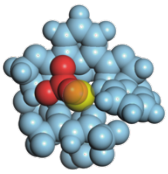 | 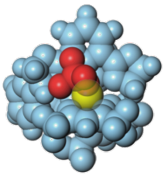 | 36.4 | 36.1 |
| (*R*)-**1**·$ReO_4^-$ | 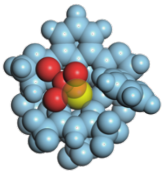 | 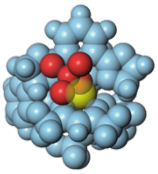 | 27.5 | 27.7 |
| (*R*)-**1**·$CF_3COO^-$ | 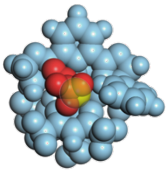 | 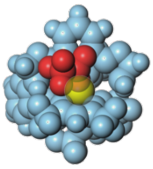 | 43.7 | 42.8 |
| (*R*)-**1**·$PF_6^-$ | 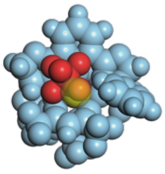 | 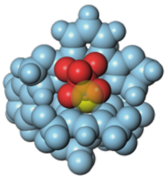 | 33.1 | 33.3 |
| (*R*)-**1**·$SbF_6^-$ | 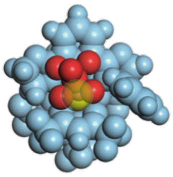 | 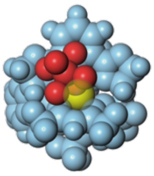 | 27.0 | 26.4 |
| (*R*)-**1**·$OSO_2CF_3^-$ | 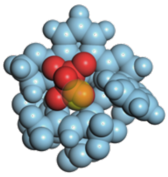 | 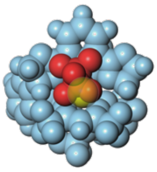 | 39.5 | 38.6 |

[a] Obtained with energy difference between the individual optimized structures of(*R*)-**1** (*A*-form and *B*-form) and anions.

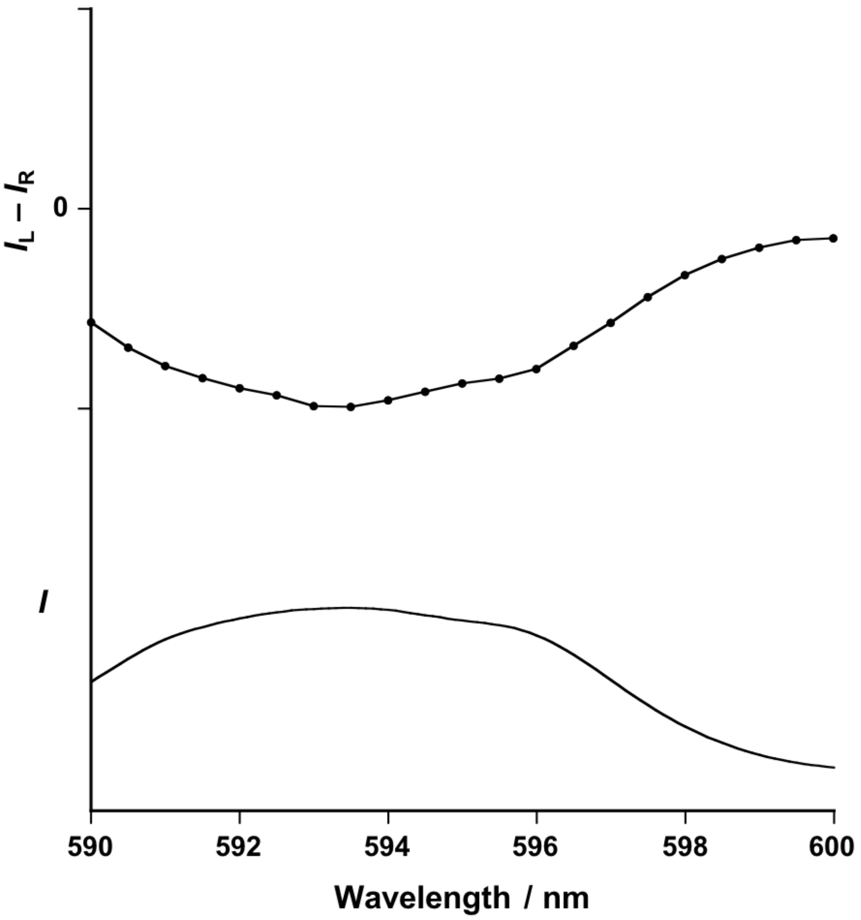


***Figure S17.*** (Top) CPL and (bottom) emission spectra of (*S*)-**1** in acetonitrile ($1.0 \times 10^{-3}$ M) with the presence of $NO_3 \cdot NBu_4$ ($7.0 \times 10^{-3}$ M) at 298 K. Excitation wavelength: $\lambda_{ex}$ = 305 nm.

**References**


1. Nishiyama, H.; Soeda, N.; Naito, T. Motoyama, Y. *Tetrahedron: Asymmetry*, **1998**, *9*, 2865.
2. Okayasu, Y. Yuasa, J. *Mol. Syst. Des. Eng.* **2018**, *3*, 66.
3. Hasegawa, Y.; Kimura, Y.; Murakoshi, K.; Wada, Y.; Yamanaka, T.; Kim, J.; Nakashima, N.; Yanagida, S. *J. Phys. Chem.* **1996**, *100*, 10201.
4. Frisch, M. J.; Trucks, G. W.; Schlegel, H. B.; Scuseria, G. E.; Robb, M. A.; Cheeseman, J. R.; Scalmani, G.; Barone, V.; Mennucci, B.; Petersson, G. A.; Nakatsuji, H.; Caricato, M.; Li, X.; Hratchian, H. P.; Izmaylov, A. F.; Bloino, J.; Zheng, G.; Sonnenberg, J. L.; Hada, M.; Ehara, M.; Toyota, K.; Fukuda, R.; Hasegawa, J.; Ishida, M.; Nakajima, T.; Honda, Y.; Kitao, O.; Nakai, H.; Vreven, T.; Montgomery, J. A., Jr.; Peralta, J. E.; Ogliaro, F.; Bearpark, M.; Heyd, J. J.; Brothers, E.; Kudin, K. N.; Staroverov, V. N.; Kobayashi, R.; Normand, J.; Raghavachari, K.; Rendell, A.; Burant, J. C.; Iyengar, S. S.; Tomasi, J.; Cossi, M.; Rega, N.; Millam, J. M.; Klene, M.; Knox, J. E.; Cross, J. B.; Bakken, V.; Adamo, C.; Jaramillo, J.; Gomperts, R.; Stratmann, R. E.; Yazyev, O.; Austin, A. J.; Cammi, R.; Pomelli, C.; Ochterski, J. W.; Martin, E. L.; Morokuma, K.; Zakrzewski, V. G.; Voth, G. A.; Salvador, P.; Dannenberg, J. J.; Dapprich, S.; Daniels, A. D.; Farkas, O.; Foresman, J. B.; Ortiz, J. V.; Cioslowski, J.; Fox, D. J. *Gaussian 09 ed.; Gaussian, Inc.: Wallingford CT*, 2009.
